# Conventional immunosuppressive therapy in severe Behcet’s Uveitis: the switch rate to the biological agents

**DOI:** 10.1186/s12886-018-0929-5

**Published:** 2018-10-05

**Authors:** Hande Celiker, Haluk Kazokoglu, Haner Direskeneli

**Affiliations:** 1 Department of Ophthalmology, Marmara University School of Medicine, Fevzi Çakmak Mah. Muhsin Yazıcıoğlu Cad. No:10 Pendik, Istanbul, Turkey; 2 Division of Rheumatology, Marmara University School of Medicine Fevzi Çakmak Mah, Muhsin Yazıcıoğlu Cad. No:10 Pendik, Istanbul, Turkey

**Keywords:** Anti-TNF-α therapy, Behçet’s uveitis, Immunosuppressive therapy, Interferon therapy, Uveitis

## Abstract

**Background:**

To report the switch rate of conventional immunosuppressive (CIS) therapies to the biological agents (BA) in patients with refractory Behcet’s uveitis (BU).

**Methods:**

In this retrospective study, clinical records were reviewed of 76 patients’ 116 eyes presenting with BU who had been treated with immunosuppressive drug therapy. Mann Whitney *U* test was used for the intergroup comparisons of parameters without normal distribution as well as calculation of descriptive statistical methods (mean, standard deviation, median, frequency and rate). Wilcoxon Signed Ranks test was used for the intragroup comparisons of parameters without normal distribution. Pearson’s Chi-Square test and Fisher-Freeman-Halton test were used for the comparisons of qualitative data.

**Results:**

Except for one, all patients were first treated with CIS regimens for BU. Thirty-one patients (41.3%) who were unresponsive to CIS regimens were switched to IFNα2a therapy. After that, eight of these cases were switched to the anti-TNF-α treatments. The presence of initial ocular complications were found to be statistically higher in BA treated patients than the CIS treated cases (*p* < 0.001). Both in CIS treated and in BA treated cases, an increase in visual acuity (VA) was observed during the last examination compared to the initial examination and was significant (*p* < 0.001 and *p* = 0.018, respectively).

**Conclusions:**

CIS treatment was found to be effective and safe, as suggested in the management guidelines for severe BU. Biological therapy was also found effective for the improvement of the VA. We observed that 58.7% of cases could be treated with strong immunosuppressive therapies, however, nearly half of the patients could have lost their VA if BAs were not existent. During the treatment course of severe cases with BU, classical therapy stage must still be protected as the first-line therapy due to the their reasonable activity and safety.

## Background

Behcet**’**s disease (BD) is a systemic vasculitis of unknown etiology manifesting mainly with oral/genital ulcers, skin lesions and uveitis. BD was first described as a distinct clinical entity by Hulusi Behcet [1]. This ubiquitous disorder is endemically higher in Turkey, Iraq, Iran, Korea, and Japan, the population derived historically from the ancient Silk Road, from the Mediterranean to the Far East and Middle Eastern countries [2]. Ocular manifestations of BD mostly include bilateral panuveitis, and retinal vasculitis with a chronic repetitive relapsing-remitting course. Systemic corticosteroids are still widely used in the therapy of ocular BD with or without conventional immunosupressive (CIS) therapies [3]. Visual impairment has been prevented in recent years with the increasing use of immunosuppressive therapies such as azathioprine (2–3 mg/kg per day), cyclosporine-A (3–5 mg/kg per day), methotrexate (7.5–20 mg per week) or mycophenolat mofetil (500 mg- 2 g/per day) [4]. Recently, interferons and other biological agents have been suggested as a second choice to CIS therapy in patients with refractory Behçet uveitis (BU) [5, 6]. Although they are shown to be superior compared to CIS therapies, biological agents are relatively new drugs and their efficacy and safety are still being investigated [7]. Use of biological agents in uveitis remains mostly limited to cases refractory to conventional treatment regimens due to their costs and our limited understanding of their long-term results [8]. Lately, in the literature, regarding BU or other uveitis treatments, most of the studies investigate the efficacy and safety profile of biological agents. Nonetheless, some of the patients with severe BU could still be treated with CIS agents. However, the reports of studies on the proportion of patients who have been changed to biological agents for the treatment of BU from CIS drugs is lacking. Thus, in this study we aimed to investigate the switch ratio from the conventional treatments to the biological therapy in patients with refractory BU in our Clinic.

## Methods

The study protocol was approved by the local Institutional Ethics Board and conducted according to the tenets of the Declaration of Helsinki. Informed consent was obtained for all procedures. In our tertiary interdisciplinary uveitis clinic, clinical records were reviewed of 76 patients’ 116 eyes presenting with severe uveitis due to BD who had been treated with immunosuppressive drug therapies from January 2008 to December 2016. Thirty-two of these cases who were treated with a biological agent (IFNα2a) were further evaluated. The patients were diagnosed on the basis of the International Study Group Criteria for BD [9]. Patients were systematically followed in both the Ophthalmology Clinic and the combined Behçet’s Clinics in our institution. Uveitis terminology was described by the Standardization of Uveitis Nomenclature (SUN) Working Group [10]. Inactive anterior uveitis was defined as 0.5 cells or less. Severe uveitis was defined as a decrease of visual acuity (VA) < 20/100, vitritis > 2+, panuveitis, or failing to respond to 1 or more conventional immunosuppressive drugs and/or requiring intermediate doses of oral corticosteroids (> 10 mg per day). Inflammation of the posterior segment was defined by the presence of retinal vasculitis, retinitis, cystoid macular edema, and papillitis. Control of intraocular inflammation reported as quiescence was documented as inactivity of anterior chamber and absence of posterior segment intraocular inflammatory signs. Remission was defined as an inactive disease for at least 3 months after discontinuation of all immunosuppressive therapy [10]. Uveitis was defined as refractory when patients were receiving the highest anti-inflammatory or immunosuppressant regimen in their lives and it was insufficient to maintain the disease under control, defined as having a history of at least 1 relapse of the disease in a year before admission that needed an escalation of the dose of oral corticosteroid or other immunosuppressive agents, including azathioprine, methotrexate, cyclosporine A, IFNα2a, infliximab, or adalimumab to control the relapse [11]. The switch criteria was defined as any patient with the diagnosis of refractory BU; using an ineffective therapy, consisting of at least 1 additional immunosuppressant drug besides corticosteroids that was not able to maintain the patient without relapses and that needed an elevation of the oral corticosteroid or other immunosuppressant dosage to control the inflammation in a year, frequency of attacks (at least one severe relapse), presence of severe uveitis complications, CIS treatment resistant leakage on fundus fluorescein angiography, level of VA (decrease of VA < 20/100 or visual loss of 2 acuity lines). We use IFNα2a as a second-line treatment in refractory BU cases. Then, we switch to anti-TNF-α therapy according to the interferon-response status or the side-effects developed.

Complete ophthalmologic examination included best corrected VA testing, biomicroscopic evaluation, tonometry, fundus examination, and optical coherence tomography (OCT) performed during the all visits. Digital color fundus photographs and fluorescein angiography (FA) were performed in all patients at least once and whenever necessary during the course.

All of the 76 patients were treated initially with corticosteroids or a CIS therapy. Thirty-two of these patients, who suffered from sight-threatening uveitis and refractory to CIS agents, were given IFNα2a therapy (Roferon-A®; Roche Pharmaceuticals, Whitehouse Station, New Jersey, US). All patients underwent evaluation by a rheumatologist at the begining of treatments. Hematologic, tyroid, and hepatic functions were analysed by rheumatologists before final inclusion into the treatment group. For treatment of patients with BU, a standardized clinical algorithm was used. In case of anterior uveitis, topical prednisolone acetate 1% every hour and topical cyclopentolate hydrochloride 1% twice per day were prescribed. If it was necessary, pulse corticosteroid therapy was used (1 g/day, for 3–5 days) for severe uveitic attacks. Systemic therapy was started generally with using corticosteroids (CS, methylprednisolone*,* 1 mg/kg/day) in combination with azathioprine (AZA, 2–3 mg/kg per day), methotrexate (MTX, 7.5–20 mg per week) or cyclosporine A (CsA, 3–5 mg/kg per day). If the dual combinations did not work, as a third-line treatment, a triple combination of CS, AZA, and CsA was initiated to the cases with refractory BU. When all these drugs were not efficacious or serious side effects were observed, treatments were switched to the biological agents. All other immunosuppressant therapies were discontinued the day before the initial IFNα2a treatment, except colchicine and topical and/or systemic CS. IFNα2a therapy was started subcutaneously at a dosage of 3.0 million IU (MIU) per day for 2 weeks as the remission induction phase. All patients received paracetamol and pheniramine maleate before and after injections to avoid flu-like symptoms At the end of the remission induction period, maintenance dose of IFNα2a was continued with 3.0 million MIU 3 times per week. Doses of steroids were tapered based on clinical improvement of uveitis and according to the severity of the systemic symptoms of BD after induction of IFNα2a therapy. In case of limitation of clinical improvement, or if steroids could not be tapered, the dosage of IFNα2a was escalated in sequences of 4.5, 6.0, and 9.0 MIU 3 times per week for each severe inflammatory attacks [12]. When IFNα2a was ineffective or untolerable adverse events were observed, anti-TNF-α (infliximab 5 mg/kg i.v.or adalimumab 40 mg sc every other week) or the other therapies were introduced.

VA was assessed in European decimals (with Snellen chart), then converted to the logarithm of the minimum angle of resolution (logMAR) for computing. NCSS (Number Cruncher Statistical System) 2007 Statistical Software (Utah, USA) program was used for the statistical analysis. During the evaluation of the study data, Mann Whitney *U* test was used for the intergroup comparisons of parameters without normal distribution as well as calculation of descriptive statistical methods (mean, standard deviation, median, frequency and rate) and Wilcoxon Signed Ranks test was used for the intragroup comparisons of parameters without normal distribution. Pearson’s Chi-Square test and Fisher-Freeman-Halton test were used for the comparisons of qualitative data.

## Results

### Patient characteristics

**Fig. 1:**
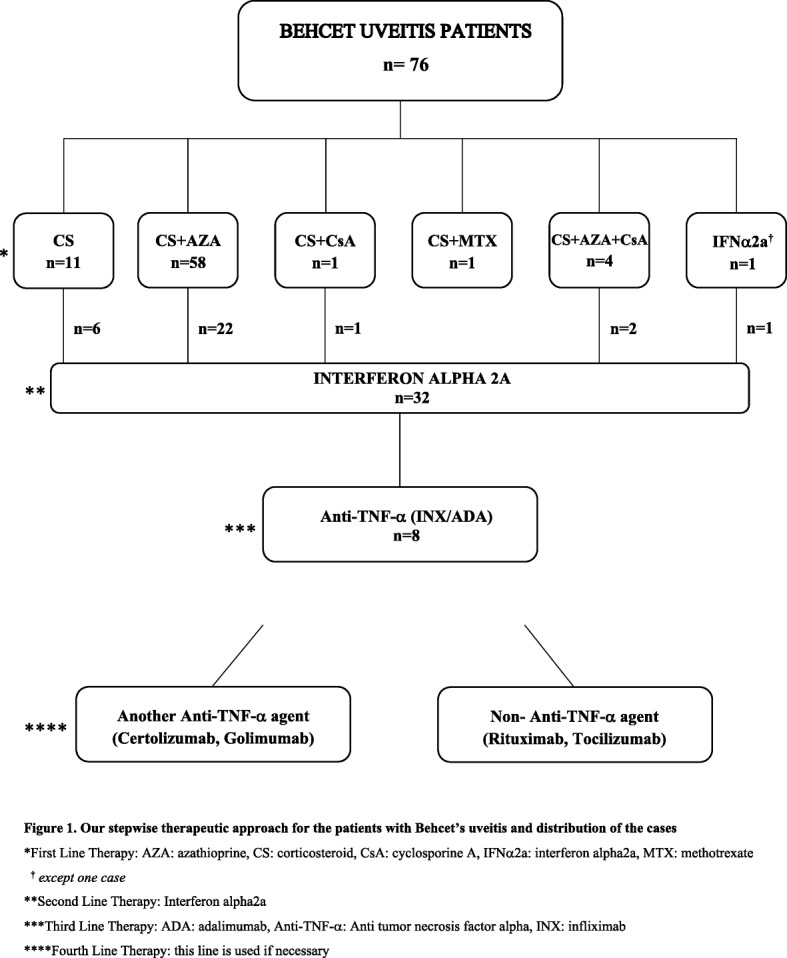
Our stepwise therapeutic approach for the patients with Behcet’s uveitis and distribution of the cases. *First Line Therapy: AZA: azathioprine, CS: corticosteroid, CsA: cyclosporine A, IFNα2a: interferon alpha2a, MTX: methotrexate. ^**†**^ *except one case.* **Second Line Therapy: Interferon alpha2a. ***Third Line Therapy: ADA: adalimumab, Anti-TNF-α: Anti tumor necrosis factor alpha, INX: infliximab. ****Fourth Line Therapy: this line is used if necessary

Seventy-six Behcet’s patients were included the study. Except for one patient, all of them were treated first with a CIS treatment regimen for BU. One patient (1.3%) was treated with IFNα2a directly due to severe systemic BD symptoms. Thirty-one patients (41.3%) who were unresponsive to CIS regimens were switched to IFNα2a therapy. The relapse features of CIS treated patients before the IFNα2a treatment were one relapse 14 (43.7%), two relapses 14 (43.7%), three relapses 2 (6.3%), and four relapses 2 (6.3%). After this step, during the therapy or after the discontinuation, non-responder eight of these cases were switched to the anti-TNF-α treatment. Forty-four cases (58.7%) continued to the CIS treatments. Treatment stepwise and distribution of the treated patients shown in Fig. 1. The mean age was 31.46 ± 8.75 years (range, 18 to 58 years). Fifty-one patients (67.1%) were men and 25 (32.9%) were women. The mean duration of follow-up was 38.70 ± 25.62 months (range, 6 to 96 months). Of BU patients, 40 (52.6%) had bilateral disease and 36 (47.4%) had unilateral ocular involvement (20 right and 16 left eyes). The most frequent type of uveitis was panuveitis (*n* = 48, 63.2%), followed by recurrent severe anterior uveitis (*n* = 16, 21.1%) and posterior uveitis (*n* = 12, 15.7%). Patients’ general characteristics and extraocular symptoms of cases are summarized in Table 1. Of 88 eyes in 116 eyes, 132 severe ocular complications were detected at the first examination. Initial ocular clinical manifestations of all patients are presented in Table 2.

**Table 1:** Patient’s descriptive characteristics and extraocular symptoms

(*n* = 76)		Mean ± sd (Min-Max)
Age (year)		31.46 ± 8.75 (18–58)
Follow-up time (month)		38.70 ± 25.62 (6–96)
		n (%)
Gender	Male	51 (67.1)
	Female	25 (32.9)
Uveitis type	Anterior	16 (21.1)
	Posterior	12 (15.7)
	Panuveitis	48 (63.2)
Laterality	Unilateral (right/left)	36 (20/16) (47.4, 26.3/21.1)
	Bilateral	40 (52.6)
HLAB51	Positive	8 (10.5)
	Negative	5 (6.6)
	Not performed	63 (82.9)
Pathergy	Positive	9 (11.8)
	Negative	18 (23.7)
	Not performed	49 (64.5)
First line therapy	CS	11 (14.5)
	CS + AZA	58 (76.3)
	CS + CsA	1 (1.3)
	CS + MTX	1 (1.3)
	CS + AZA + CsA	4 (5.3)
	IFNα2a	1 (1.3)
Extraocular symptoms	Recurrent oral ulcers	76 (100)
	Genital ulceration	52 (68.4)
	Folliculitis	47 (61.8)
	Arthritis	41 (53.9)
	Erythema Nodosum	19 (25.0)
	CNS involvement	9 (11.8)
	Thrombophlebitis	4 (5.2)

*AZA* azathioprine, *CS* corticosteroid, *CsA* cyclosporine, *IFNα2a* interferon alpha2a

**Table 2:** Distribution of ocular manifestations at initial examination

	n (%)
Ocular Complications	
None	28 (24.1)
Vasculitis	60 (51.7)
Macular edema	23 (19.8)
Retinitis	18 (15.5)
Papillitis	18 (15.5)
Opaque media	4 (3.4)
Vitreoretinal Adhesions	2 (1.7)
Lamellar Macular Hole	2 (1.7)
Epiretinal membrane	2 (1.7)
Keratitis	1 (0.9)
Optic atrophy	1 (0.9)
Recurrent hypopion	1 (0.9)

Multiple complications were seen in one eye

### Descriptive characteristics of patients

Between the CIS treated patients and biological agent-treated cases, there were no statistically significant differences in terms of mean age, duration of BD, gender, pathergy positivity, presence of HLA-B51, and first attack treatment approach (*p* > 0.05). Follow-up periods of the biological agent-treated patients were significantly longer than the conventional treatment patients (*p* < 0.001). In the biological agent-treated group, the percentage of panuveitis was found to be statistically higher than the conventional drug treated patients (*p* < 0.001). Presence of initial ocular clinical complications were also significantly higher in biological agent-treated patients compared to conventional drug treated cases (*p* < 0.001). The bilaterality ratio of the biological agent-treated patients were significantly higher than the conventional drug treated patients (*p* < 0,05).

### Visual acuity

**Fig. 2:**
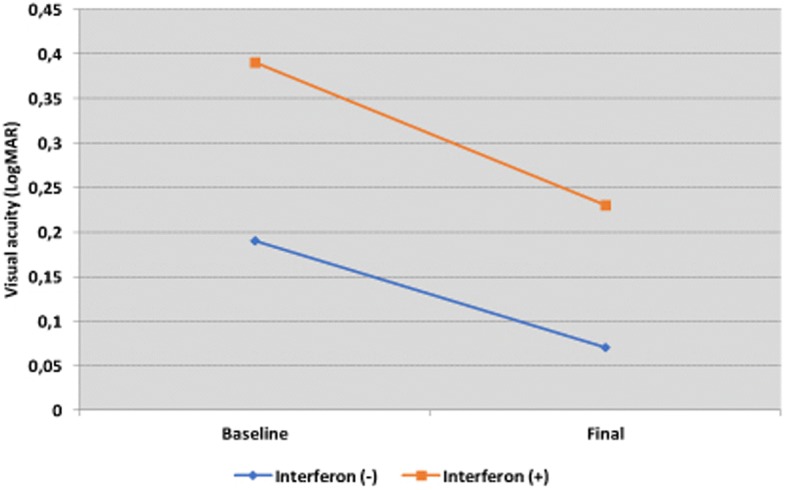
Distribution of LogMAR visual acuity of conventional agent treated patients and Interferon alpha 2a  treated cases

Both groups (CIS and BA patients) had significant improvement in VA at last visit when compared to baseline (*p* = 0.003, *p* = 0.030, respectively), although VA scores were higher in the CIS group for both time points (*p* < 0.001). In biological agent-treated cases, the increase in VA observed during last examination compared to initial examination was also determined to be statistically significant (*p* = 0.018). Distribution of LogMAR VA of two treatment groups is shown in Fig. 2. After the discontinuation of IFNα2a treated 3 cases who was started anti-TNF-α agents during this period, significantly loss of vision (logMAR ≥2.0) occured in 3 eyes due to the severe former relapses of disease and in one eye rhegmatogenous retinal detachment also happened. These cases had experienced severe uveitis attacks during the all treatment period, before anti-TNF-α agents was started.

### Adverse effects

We did not observe severe side effects related to CIS therapy. Adverse effects related to IFNα2a therapy occurred in the following frequencies: flu-like syndrome associated with myalgia and fever (at the initiation phase of the treatment) (100%, *n* = 32), fatigue (12.5%, *n* = 4), loss of weight (6.25%, *n* = 2), mild leukopenia (> 2.000/μl) (6.25%, *n* = 2), elevation of serum liver enzymes (alanine transaminase (ALT, range 0–55 U/L), aspartate transaminase (AST, range 5–34 U/L)) (6.25%, *n* = 2), severe diarrhea (3.1%, *n* = 1), dissemine and intractable fibromyalgia (3.1%, *n* = 1), loss of appetite (3.1%, *n* = 1), hair loss (3.1%, *n* = 1), dryness of the mouth (3.1%, *n* = 1). Flu-like syndrome associated with myalgia and fever was well controlled with premedication using paracetamol and pheniramine maleate in all patients. Depression and aggressiveness were no observed. IFN-α2a therapy was discontinued due to adverse events in two patients (severe diarrhea and excessive weight loss). Nevertheless, these patients were non-responder to IFNα2a therapy at the same time (despite escalating of IFNα2a treatment dosages). As a consequence, their treatment was changed to anti-TNF-α agents. During the anti-TNF-α agent treatment (adalimumab), one patient have suffered for tuberculosis infection. The drug was discontinued, the patient is still being followed by us and infectious disease specialists.

## Discussion

BD is a multiorgan disease characterized by an immune-mediated occlusive vasculitis [4]. Although BD represents a multisystemic disease, ocular involvement may reduce quality of life more than other complications of the disorder for many Behcet’s patients. The use of advanced immunosuppressive drugs have virtually prevented the loss of vision in many patients with BU. Nonetheless, due to uncontrolled progression of the disease, a substantial proportion of patients with BU cannot be treated sufficiently with these conventional agents. In 1986, Tsambos introduced for the first time the successful interferon treatment of three patients with BD, as herpes simplex virus type 1 was regarded to be involved in the etiopathogenesis in BD [13]. TNF-α inhibitors have also been shown to be effective and safe for the treatment of various diseases like rheumatoid arthritis, ankylosing spondylitis, juvenile idiopathic arthritis, Crohn’s Disease, sarcoidosis and also uveitis. We intended to report our 8-year experience with the treatment of patients with BU. We previously evaluated risk factors of the patients who needed to the biological agents. Being young was detected as a poor prognostic factor in the multivariable analyses (unpublished data, Celiker et al., manuscript in review). Our main purpose in the present study was to report our renunciation from the conventional agents therapy in patients with BU and also the switch ratio of powerful immunosuppressants to the biological agents in our clinic. We did not aim to make direct comparisons between two patient groups, as a selection bias was present for the biological treatment group. Due to this selection, the percentage of panuveitis and presence of initial ocular clinical complications were found to be statistically higher in biological agent-treated patients than with CIS group. As known, these parameters are predictors of probable advance treatment requirements in the future.

CIS agent’s activity and safety profile have been known for many years, therefore studies on these drugs are no longer published. Recently, most of the studies have been evaluating the efficacy and safety of IFNα2a and anti-TNF-α agents in BU. We believe that the studies which are related to CIS agents are still valuable, since we still are able to treat many patients with BU with these conventional drugs. In the present study, we could treat more than half of our cases of severe BU with these agents.

In the present study, we obtained accomplished VA results from the patients who were treated with conventional agents. In the biological agent-treated cases, the increase in VA during last examination was also acquired. As a result of assessment of VA in the whole cases, at the initial and the last examination, the VA of patients who were treated with immunosuppressant agents was determined to be statistically significantly higher than the patients treated only with biological agents. Due to the existence of more severe ocular inflammation in biological agent-treated patients than the others, this was an expected result. Therefore, both treatment modalities demonstrated powerful efficacy in the treatment of patients with BU.

For our country, we calculated the costs for treatment regimens for a patient with BU of 60 kg of body weight. For anti-TNF-α treatment (adalimumab), the cost will be US $15,600 (induction and maintenance dose regimen: 40 mg/0.8 ml two times per month) per year. For IFNα2a (Roferon-A®; Roche Pharmaceuticals), the cost will be US $1485/year (induction and maintenance dose regimen: 3.0 MIU 3 times per week). For the same patient, AZA (Imuran® Glaxo Smithkline Pharmaceuticals Ltd., 150 mg per day) + CsA (Sandimmun Neoral® Novartis Pharmaceuticals Ltd., 200 mg per day) combination treatment cost will be US $882 per year. In comparison, there is a significant difference in terms of costs of the drugs. Of course, drug choice should always be considered in favor of the patient, however, the cost-effectivity should also be taken into consideration when selecting the eligible agent. Kötter et al. emphasized that there is a need for studies with IFNα2a using standardized outcome measures and pre-and post-treatment observation periods in this era of evidence-based medicine [7]. Besides the efficacy of a drug, cost-effectiveness should be compared with standard immunosuppressants to determine its hierarchy in the treatment of BD [7]. In our opinion, in the fashion of the present study, the reports about comparing the cost-effectiveness of both treatment regimens may be important for the establish of the BU therapy algorithm.

In some uveitis clinics, due to their well documented intense effects to control BU, anti-TNF-α agents’ are being used as a second line therapy after single or combination CIS treatment. However, according to our stepwise therapeutic approach, anti-TNF-α agents are used as a third line therapy for BU patients who fail or do not tolerate second line IFNα2a treatment. Furthermore, it is also important that each clinic’s treatment algorithms should be determined according to the healthcare system of their own country.

## Conclusions

CIS treatment was effective and safe as has been known for a long time in the management of BU. Biological therapies were also found to be effective for the improvement of the VA. Comparative studies of the two treatment modalities may lead to bias due to the fact that patients in the biological-agent group do not respond to the conventional treatment. However, in our opinion, despite the new biological agents, it is still important to know how often we can treat our patients with classical immunosuppressive drugs. In the present study we observed that while 58.7% of cases could be treated with strong immunosuppressive therapies, nevertheless, 41.3% of patients had to be treated with biological agents. Namely, according to the outcomes of the present study, nearly half of the our patients could have lost their VA if biological agents have not been existent. The cost-effectiveness of the biological agents compared with the standard immunosuppressive drugs also should be considered during the selection of appropriate treatments, particularly as a first-line agent. In our opinion, during the treatment course of severe cases with BU, in the treatment algorithm, the classical therapy stage must be protected as a first-line therapy due to the strong activity and safety. Nonetheless, as known by all BD specialists, BU can lead to irreversible visual loss, especially in younger individuals, thus, the choice of the appropriate prompt treatment is essential for these patients. Therefore, in our opinion, there is a need for more controlled, randomized studies with conventional strong immunosuppresive drugs and biological agents using standardized outcome measures, and their efficacy and cost-effectiveness should be compared to determine their hierarchy in the treatment of BU.

## Abbreviations

ADA: Adalimumab
ALT: Alanine transaminase
Anti-TNF-α: Anti tumor necrosis factor alpha
AST: Aspartate transaminase
AZA: Azathioprine
BA: Biological agents
BD: Behcet**’**s disease
BU: Behcet’s uveitis
CIS: Conventional immunosuppressive
CS: Corticosteroid
CsA: Cyclosporine A
FA: Fluorescein angiography
IFNα2a: Interferon alpha2a
INX: Infliximab
MTX: Methotrexate
OCT: Optical coherence tomography
SUN: Standardization of Uveitis Nomenclature
VA: Visual acuity
